# A novel spatially resolved interactance spectroscopy system to estimate degree of red coloration in red-fleshed apple

**DOI:** 10.1038/s41598-021-01468-z

**Published:** 2021-11-09

**Authors:** Xujun Ye, Tamaki Doi, Osamu Arakawa, Shuhuai Zhang

**Affiliations:** Faculty of Agriculture and Life Science, Hirosaki University, Aomori, 036-8561 Japan

**Keywords:** Optical sensors, Optoelectronic devices and components, Optical spectroscopy, Near-infrared spectroscopy, Spectrophotometry, Imaging and sensing, Near-infrared spectroscopy, Spectrophotometry

## Abstract

Reliable information about degree of red coloration in fruit flesh is essential for grading and sorting of red-fleshed apples. We propose a spatially resolved interactance spectroscopy approach as a new rapid and non-destructive technique to estimate degree of red coloration in the flesh of a red-fleshed apple cultivar ‘Kurenainoyume’. A novel measurement system was developed to obtain spatially resolved interactance spectra (190–1070 nm) for apple fruits at eight different light source-detector separation (SDS) distances on fruit surface. Anthocyanins in apple were extracted using a solvent extraction technique, and their contents were quantified with a spectrophotometer. Partial least squares (PLS) regression analyses were performed to develop estimation models for anthocyanin content from spatially resolved interactance spectra. Results showed that the PLS models based on interactance spectra obtained at different SDS distances achieved different predictive accuracy. Further, the system demonstrated the possibility to detect the degree of red coloration in the flesh at specific depths by identifying an optimal SDS distance. This might contribute to provide a detailed profile of the red coloration (anthocyanins) that is unevenly distributed among different depths of the flesh. This new approach may be potentially applied to grading and sorting systems for red-fleshed apples in fruit industry.

## Introduction

Red-fleshed apples are welcomed by consumers because of their unique flesh colors and additional health benefits offered by more antioxidants present in the flesh. ‘Kurenainoyume’, literally called ‘crimson dream’, is a new red-fleshed apple cultivar bred by Hirosaki University, Japan^1^. Like many other red-fleshed apple varieties, ‘Kurenainoyume’ suffers from significant difference in flesh red coloration among individual fruits. The degree of red coloration in the flesh cannot be known unless the fruit is cut. Such destructive method can be used to check fruit samples, but the checking results for only a limited number of randomly selected samples cannot accurately reflect the degree of red coloration in other fruits even they are from the same harvest batch. Therefore, there is a need to develop an accurate, rapid and non-destructive technique for determining the degree of red coloration for individual fruits.

Optical spectroscopy is one of the most direct and common non-destructive methods for evaluating the internal quality of agricultural products^2–4^. By combining it with various chemometric techniques, optical spectroscopy has been extensively examined for determination of various internal quality properties in fruits and vegetables^5–8^. A lot of various types of equipment based on this technology have been developed and applied in fruit sorting and grading lines and packing houses. Adoption of this non-destructive technique saves a great deal of manual labor and thus greatly increases the efficiency of fruit sorting and grading in factory lines^9,10^. However, most of the equipment used in practice as well as the prototypes developed in recent studies focused on the assessment of sweetness^11,12^, ripeness^13,14^, the detection of bruise^15,16^ and hollow heart or cavity^17,18^ in the inner parts (internal quality), and the appearance color of fruit skin (external quality)^19,20^, which are the commonly used parameters for fruit sorting and grading. These techniques sort and grade the fruits based on the estimated values of the property parameters, which are selected as criteria for fruit sorting and grading. However, little research has been reported on fruit sorting and grading of red-fleshed apples based on the degree of red coloration in the flesh using an optical spectroscopic approach.

In our previous work, an interactance spectroscopy device was developed to collect interactance spectra to investigate the red coloration in the red-fleshed ‘Kurenainoyume’ apple fruits^21^. Unlike the most commonly used reflectance and transmittance spectroscopy, the interactance spectroscopy collects the so-called interantance spectra by a measurement setup that allows the incident beam to penetrate into the surface and interact with the sample, and finally collects the scattered beam from a point that is different from the point of incidence on the sample’s surface^22–24^. In interactance spectroscopy, the interactance is obtained after the beam interacts with the inner parts of the material over a certain light transmit path, therefore its intensity embodies more information about the physical structure as well as the biochemical composition of the inner parts of the sample than the reflectance that is collected on the same point of light source. In addition, the transmittance can be obtained only for samples that the incident beam could pass through; therefore, it is almost infeasible to apply it to the big and solid flesh fruits like apples. Hence, the interactance spectroscopic approach has been used to replace the reflectance and transmittance approaches to determine the contents of particular compounds in various samples^25–27^.

The device developed in our previous work obtained interactance spectra with a constant distance between the light source and the detector. Several predictive models were developed for estimating the degree of red coloration in the flesh, with different levels of accuracy achieved. These results suggest the interactance technique combined with the developed models might provide a new rapid and non-destructive approach for assessing the internal quality of red-fleshed apples^21^. As a further step of this research, based on the previous work we recently developed a novel interactance spectroscopy system to obtain spatially resolved interactance spectra from apple fruits. The new system could obtain interactance spectra with a detector, whose distance from light source could be adjusted to eight specified distances. In this study, we employed the new system to investigate the feasibility of spatially resolved interactance spectroscopy for estimating the degree of red coloration in the flesh of ‘Kurenainoyume’ apples.

## Rationale

Light is used as a common tool to noninvasively extract useful information on internal properties of the materials in many research fields such as pharmaceutical and biomedical analysis^28,29^, quality inspection and grading of agricultural and food products^30,31^. Usually, the light is emitted at a certain position and collected at the same position or at a certain distance from the light beam. The photodetector can be placed on the same, opposite or any other side of the external surface of the material, depending on the shape and thickness of the material under investigation. The behaviors of light entering the surface of the material have been extensively studied in the literature^32–34^. Both theoretical and experimental research found that the light collected strongly depends on the migration path distance and absorption by the material, and the penetration depth increases as the migration path distance increases or absorption decreases^35,36^. These results suggest that the light collected by the photodetector carries information on the optical properties of the material encoded along photons migration paths, and therefore can provide useful information for measurements of internal physical and biochemical properties of the materials.

In interactance spectroscopy, the light is collected with a detector placed at a certain separation distance from the light source on the same side of the external surface of the material^22–24^. This approach is strongly dependent on how much the light interacts with the internal matters during the light migration^37^. The separation distance between the light source and the detector, also abbreviated as Source-Detector Separation (SDS) distance, is a key factor that influences how much information on internal properties can be collected. For this reason, to increase as much as possible the detectability of internal properties the optimal SDS distance is usually the main quantity investigated in interactance spectroscopy^38–40^. Particularly, when the internal properties are not evenly distributed in the material, the optimal SDS distance might be different for internal properties at different depths, and they can be potentially identified for individual depths reached by the migrating photons.

**Figure 1:**
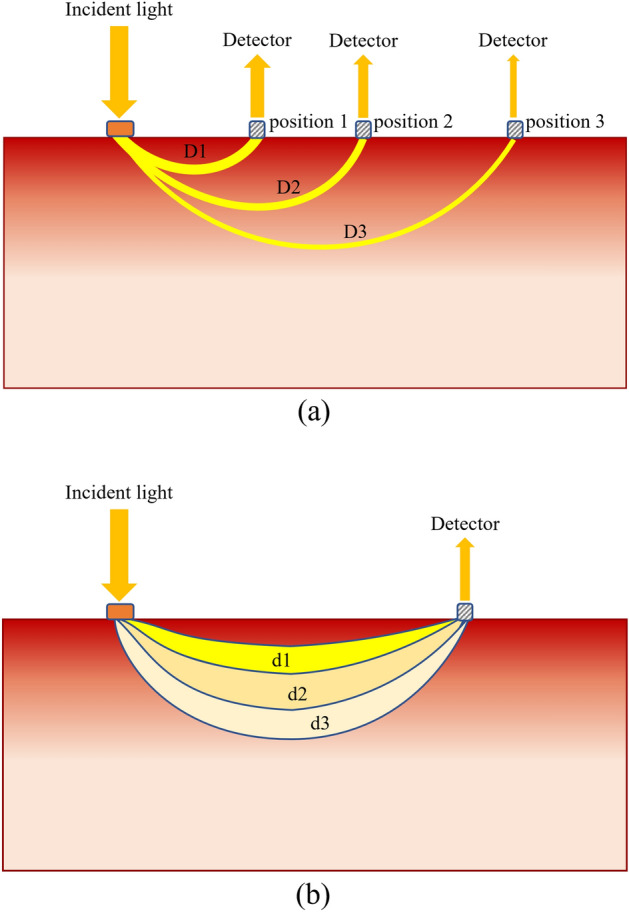
Graphical illustration of the pathways of light and their intensities in fruit flesh. (**a**) The light has a longer path length, and its intensity becomes weaker as it reaches farther away from the light source; (**b**) The light has a longer path length as it reaches greater depths, showing a banana-shaped path. Mechanism of multiple detectors at different positions on samples might acquire information needed for analysis of internal properties of fruit flesh at different depths.

Apparently, for a longer SDS distance, the photons migrate longer lengths, and the photons detected becomes fewer because more light-matter interactions (penetration and absorption) occur during a longer light migration path (Fig. 1a). In addition, previous research indicates that, for a given SDS distance, the photons that migrate deeper have longer path lengths, thus forming a banana-shaped path^41^, as illustrated in Fig. 1b. Because the photons follow the banana-shaped path back to the surface of the material, these photons measured using photodetectors contain combined optical properties for all depths reached by the migrating photons. As mentioned above, because the photons that migrate deeper have longer path lengths, there might be more light-matter interactions in the deeper layers than those immediately beneath the surface of the material. Therefore, we hypothesize that such an interactance spectroscopic approach might be more suitable to detect the internal properties of the deeper layers of the material. A key issue in this approach is to identify the optimal SDS distance for the layer at a specific depth. In this study, we developed a novel measurement system to obtain spatially resolved interactance spectra and used it to identify the optimal SDS distance for detecting the red coloration in the flesh of the red-fleshed ‘Kurenainoyume’ apple cultivar.

## Results

### Red coloration and anthocyanin content in apple skin and flesh

**Figure 2:**
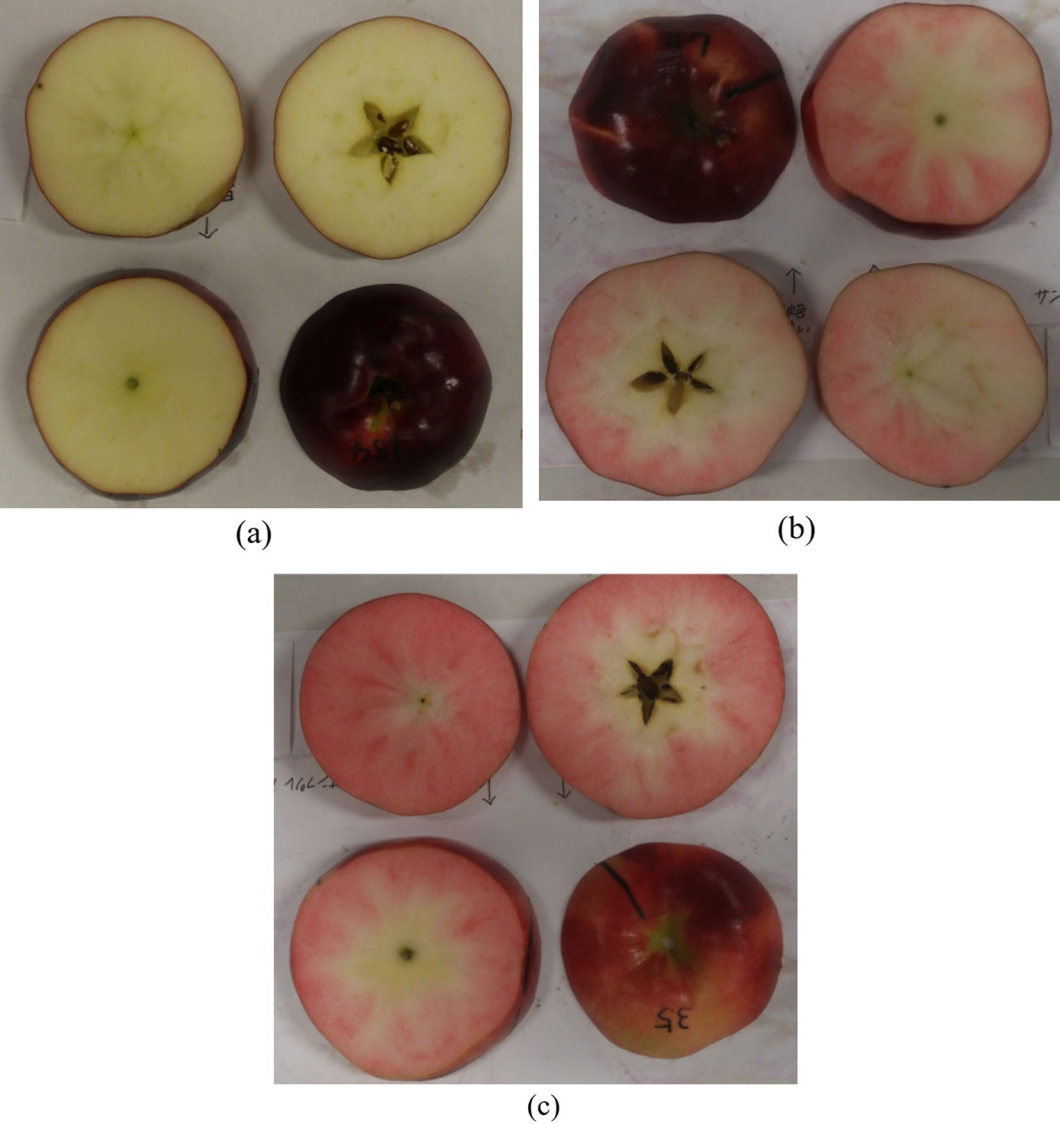
Red coloration in red-fleshed apple ‘Kurenainoyume’. Three samples with low (**a**), medium (**b**) and high (**c**) levels of red coloration in the flesh were selected. The skin appearance, 3 flesh slices for each sample were illustrated. It was found that the skin color does not exactly reflect the color of the flesh.

The degree of red coloration in the skin and flesh showed a large variation among different fruit samples (Fig. 2). Further, it was also found that the skin color did not exactly match the color of the flesh. For example, the fruit sample (Fig. 2a) had a fruit skin in dark red color, but the flesh showed the same white color as the common, white-fleshed apples. In contrast, the fruit sample (Fig. 2c) had a light red skin color, but the flesh was highly pigmented. This suggests the difficulty to discriminate the flesh color simply by visual inspection of the appearance of the fruit skin.

**Figure 3:**
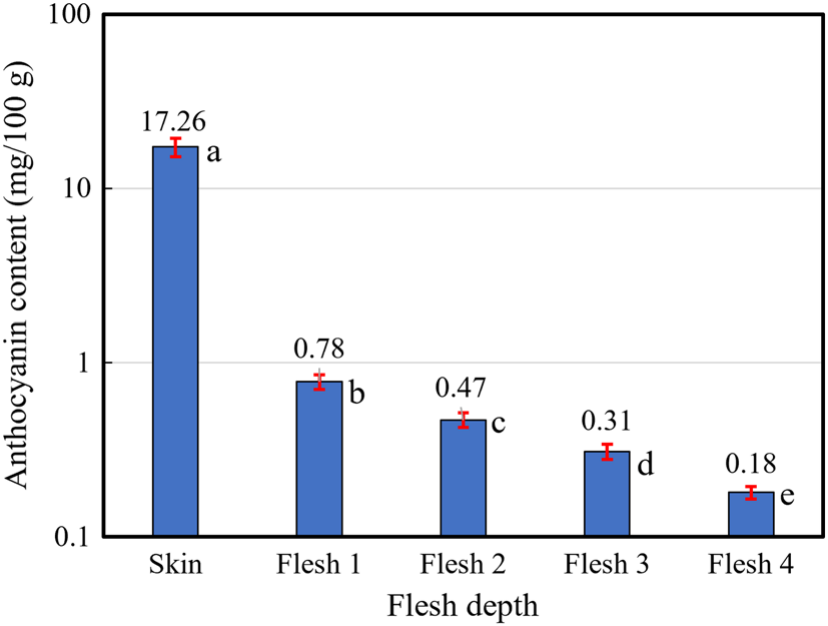
Anthocyanin contents in samples of skin and fleshes at different depths. Averages and standard errors of anthocyanin contents were indicated on a logarithmic scale. There were statistically significant differences between skin and flesh depth (*p* = 0).

The anthocyanin contents in skin and flesh at each individual depth were summarized in Fig. 3. There were statistically significant differences between flesh depth (*p* = 0). The anthocyanins in skin (17.26 mg/100 g) were more than several ten-folds higher than those in the whole flesh (average of all flesh layers: 0.44 mg/100 g). The anthocyanin content in individual flesh layer decreased with flesh depth. The average anthocyanin content in the whole flesh ranged from 0.094 to 1.416 mg/100 g for the fruit samples, with an average of 0.417 mg/100 g and standard deviation of 0.270 mg/100 g, respectively, showing a tremendous difference in anthocyanin content among fruits, which is responsible for the different degree of red coloration in the flesh among fruits.

### Correlations between anthocyanin contents in skin and fleshes at different depths

Table 1 shows the correlation matrix for skin and all fleshes at different depths included in this study. Skin showed a low correlation with all fleshes at different depths, with the correlation coefficient (*r*) less than 0.5 except the outer flesh (Flesh 1) immediately beneath the skin (*r* = 0.503). This confirmed that the skin color does not necessarily reflect the color of the internal flesh, particularly the inner flesh that is relatively far away from the skin (Fig. 2). Further, high correlations were found between neighboring flesh layers at different depths (*r* > 0.8). The degree of correlation decreased when the two flesh layers are apart from each other. However, Flesh 1 and Flesh 3 showed the highest correlation among all skin and fleshes at different depths (*r* = 0.894).

**Table 1:** Correlations between anthocyanin contents in samples of skin and fleshes at different depths.

	Skin	Flesh 1	Flesh 2	Flesh 3	Flesh 4
Skin	1.000				
Flesh 1	0.503**	1.000			
Flesh 2	0.397**	0.876**	1.000		
Flesh 3	0.475**	0.894**	0.841**	1.000	
Flesh 4	0.479**	0.700**	0.602**	0.831**	1.000

Person’s correlation coefficients were indicated. **Correlation is significant at the 0.01 level (2-tailed).

### Characteristics of spatially resolved interactance spectra

**Figure 4:**
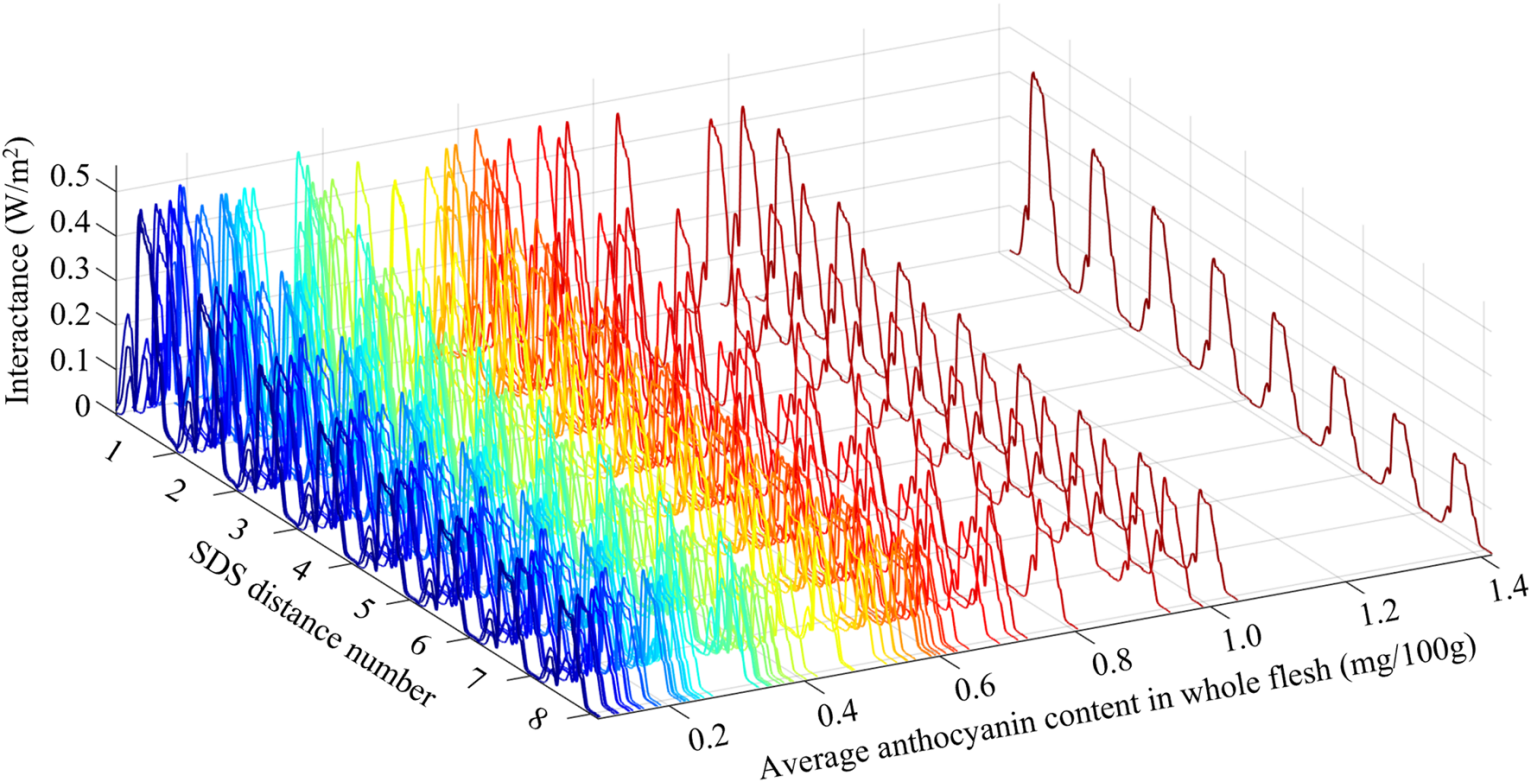
Spectral characteristics of interactances obtained at different SDS distances for all fruit samples. The larger in SDS distance number, the farther the detector from the light source. For each SDS distance number, the interactances for the wavelengths ranging from 500 to 1070 nm were illustrated. The spectra were sequentially ordered in terms of the anthocyanin content of the whole flesh, which was calculated by averaging the anthocyanin content of each individual flesh.

The spatially resolved interactance spectra were collected by the detector at eight different SDS distances. Because no or little interactance was detected in the short wavelength range, the interactances only for the wavelength range from 500 to 1070 nm were illustrated. These interactances collected at each SDS distance were first sequentially connected into one single signal spectrum for each fruit, and then the spectra for all fruit samples were summarized, as illustrated in Fig. 4. It is found that the detector at each SDS distance obtained a different interactance intensity for the same sample. In comparison, distances close to the light source have a higher interactance and its intensity decreases as the detector gets farther away from the light source. This indicates that it becomes more difficult for the light to travel a longer distance (due to its interaction with and absorption by various elements in the flesh), and therefore only a part of light entering the flesh can be detected by the sensor after the long travel process. Furthermore, a close look at the interactances at the same distances for different fruits indicated that overall, the fruits with higher levels of anthocyanin content had a lower interactance intensity than those with lower anthocyanin contents, due to different levels of light absorption by anthocyanins in fleshes.

**Figure 5:**
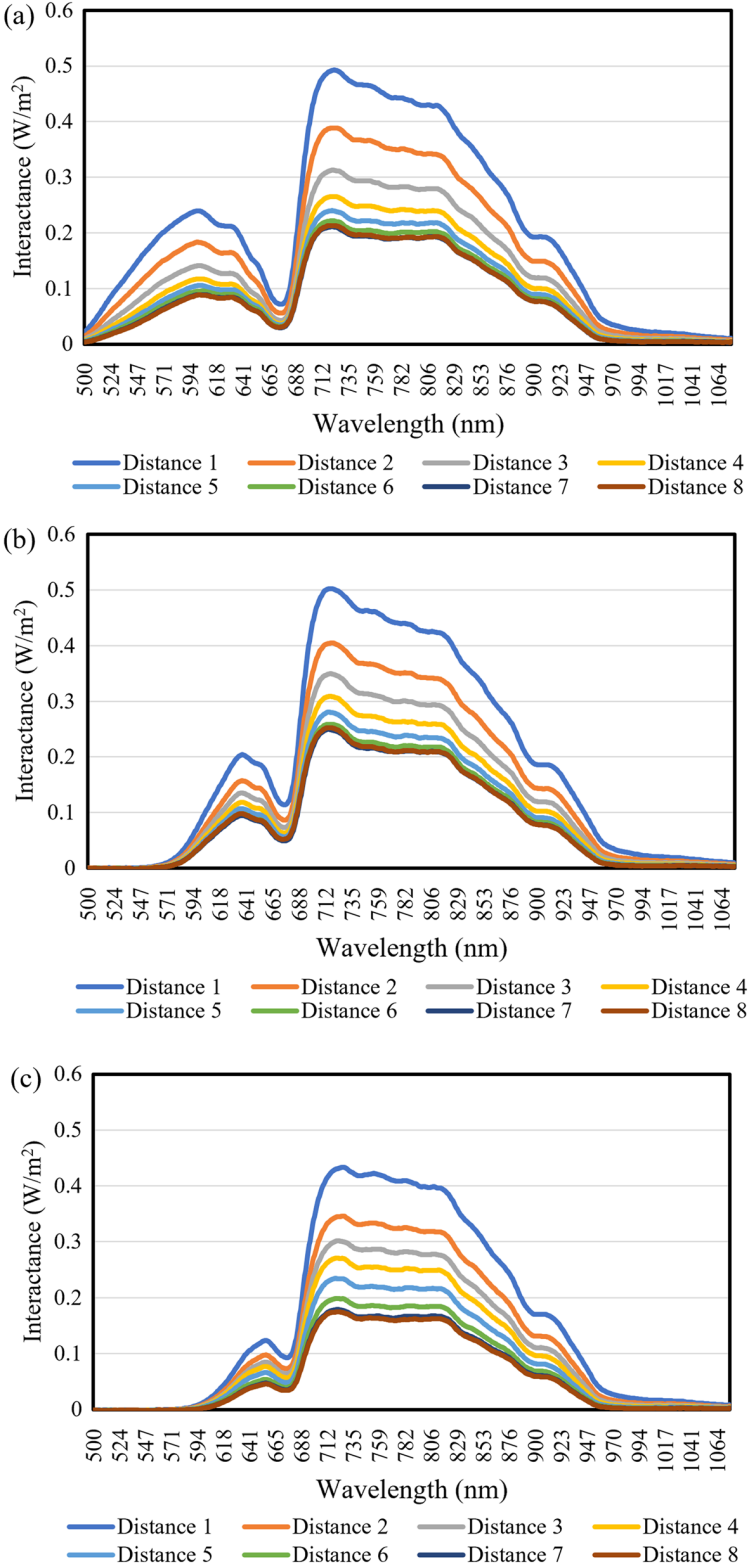
Spectral characteristics of interactances obtained at different positions for fruit samples with different levels of anthocyanin contents. The averaged whole flesh anthocyanin contents of the low (**a**), medium (**b**), and high (**c**) anthocyanin content fruits were 0.094, 0.5513 and 1.416 mg/100 g, respectively.

The spatially resolved interactance spectra showed significant differences in the signal intensities of interactances among different specific SDS distances, though all specific SDS distances showed a similar pattern along the wavelength range (Fig. 5). The specific SDS distance number 8 (the center of the device) showed the lowest interactances, and the interactances increased gradually (the spectra shifted upwards) as the distances of the detector’s specific positions from the light source became closer (a smaller SDS distance number). Furthermore, the overall spatially resolved interactances of the fruit sample with a high anthocyanin content were lower than those of the fruit with a lower anthocyanin content, particularly within the 500–660 nm wavelength range. In addition, the peaks of interactances shifted from a shorter wavelength to a longer one with the increase of anthocyanin content in apple flesh (fruit a: 590 nm, fruit b: 630 nm, fruit c: 650 nm). These results suggest that the spatially resolved interactances in these wavelength ranges may provide useful information about the anthocyanin content in apple flesh.

### PLS models

The average interactances of eight different SDS distances were used to develop PLS models for estimating anthocyanin content in skin and individual flesh. Three types of analysis of variance (ANOVA) conducted to compare the predictive accuracy of the models were summarized below.

#### Two-way ANOVA of model accuracy for skin and flesh at different depths

A two-way ANOVA was conducted that examined the effect of SDS distance and flesh depth on model prediction accuracy (*R*^2^). Simple main effects analysis showed that both SDS distance (*p* = 0) and flesh depth (*p* = 0) significantly influenced the model prediction accuracy. Moreover, there was a statistically significant interaction between the effects of SDS distance and flesh depth on model prediction accuracy (*p* = 0) (Table 2).

**Table 2:** Two-way ANOVA results of main and interactive effects of SDS distance and flesh depth on model prediction accuracy.

Source	SS	df	MS	F	p-value
Detector distance	0.221	7	0.0316	13.25	0
Flesh depth	84.5629	4	21.1407	8870.08	0
Interaction	0.575	28	0.0205	8.62	0
Error	4.6714	1960	0.0024		
Total	90.0303	1999			

**Figure 6:**
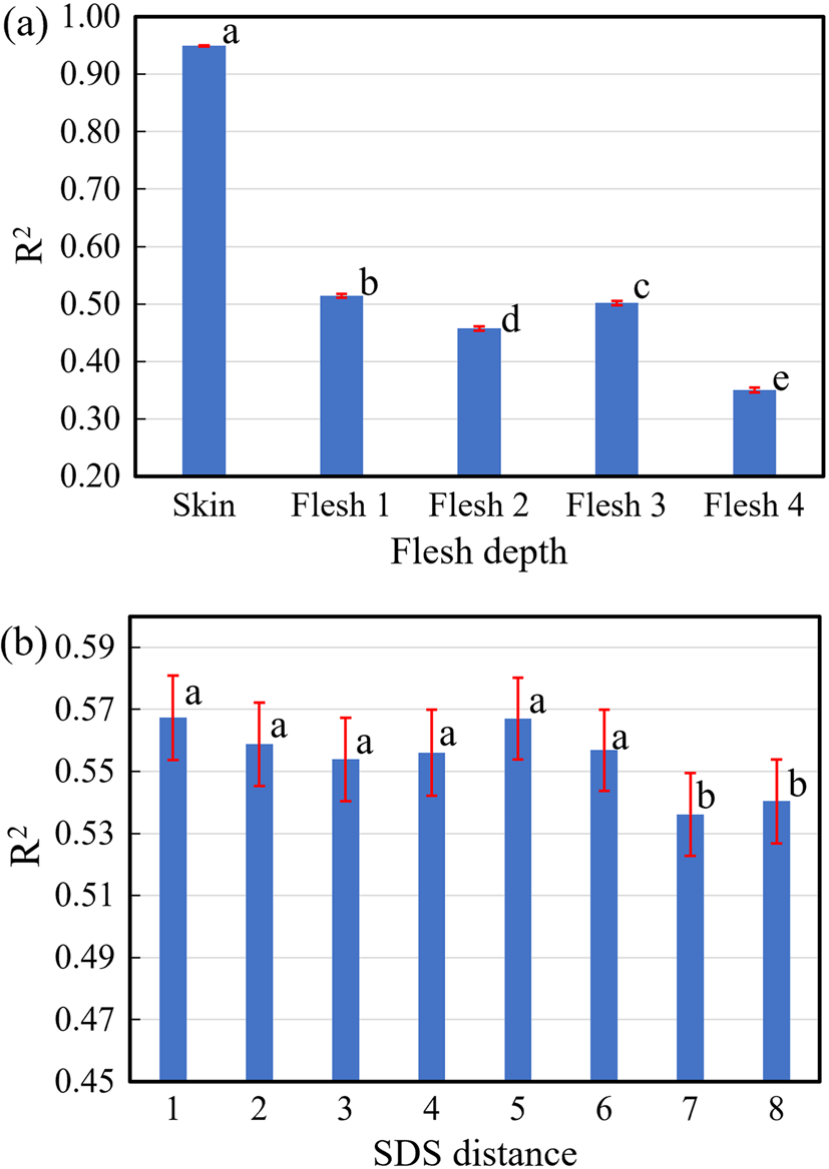
Average (± SE) *R*^2^ for PLS models based on different flesh depths (**a**) and SDS distances (**b**). Flesh depth significantly influenced the prediction accuracy (*p* < 0.001). Significant differences were observed between short (1 through 6) and longer (7–8) detector distances (*p* < 0.001).

Flesh depth significantly influenced the prediction accuracy (*p* < 0.001) (Fig. 6a). The models for skin obtained a very high predictive accuracy (*R*^2^ > 0.9). The predictive accuracy of models for all flesh depths decreased sharply compared to that of the skin model. This indicates that it is much more difficult to detect the properties of flesh than the skin. The model performance became poorer from the outer layer Flesh 1 to Flesh 2, however, it again improved for the Flesh 3, and finally reached the lowest for the most inner layer Flesh 4.

In addition, significant differences were observed between shorter (1 through 6) and longer (7–8) SDS distances (*p* < 0.001) (Fig. 6b). Although there was no statistically significant difference between SDS distances 1 through 6, the models obtained at distance 5 achieved the highest predictive accuracy. This might be attributed to the fact that more interactions occur between scattered light and fruit tissues when light passes through a longer distance within the flesh of a fruit, and these interactions enable the collection of interactance spectra with more information about the internal properties of fruits. Nevertheless, the SDS distances 7–8, which have even longer light SDS distance, demonstrated the lowest prediction accuracy. This suggests that when the SDS distance exceeds a certain distance limit, the interactance detected might become much less informative because most of the light is absorbed by the flesh and thus cannot reach the detector.

#### One-way ANOVA of model accuracy for whole flesh

**Figure 7:**
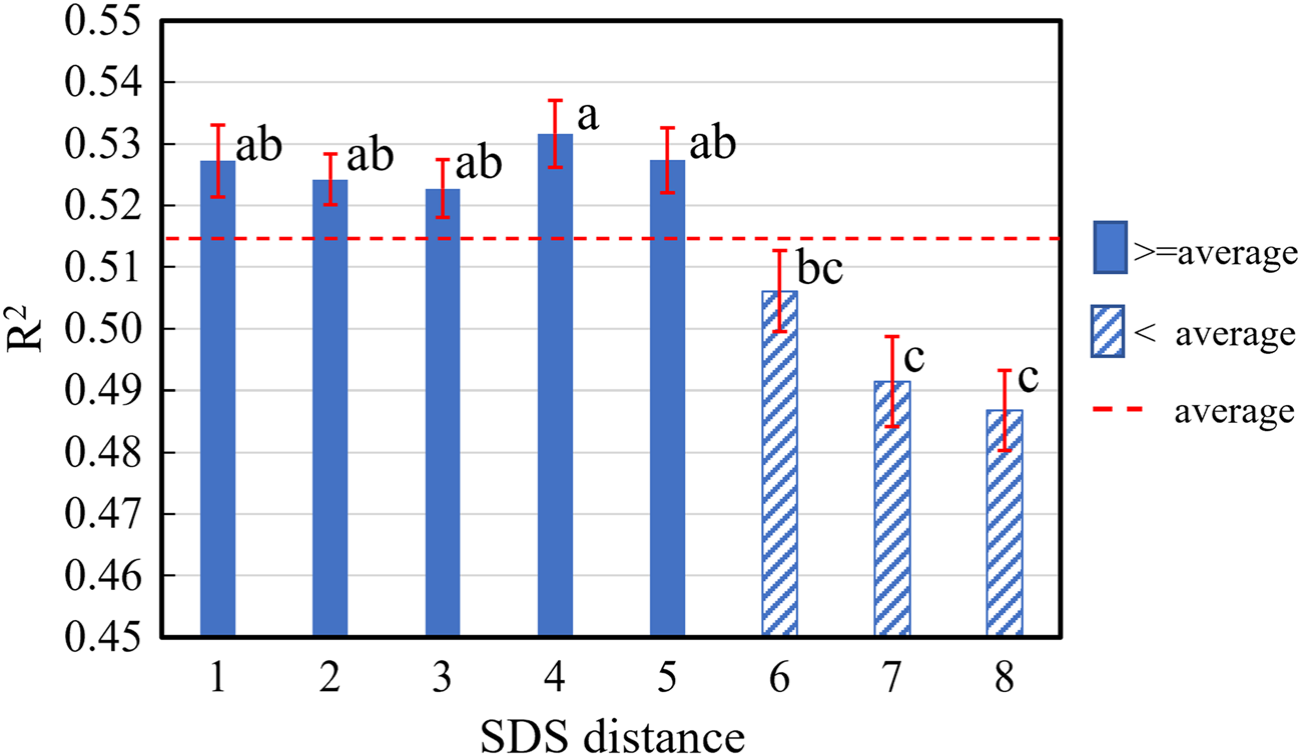
Average (± SE) R^2^ for PLS models for the whole flesh based on interactances collected at different SDS distances. Significant differences were observed between short (1 through 5) and longer (6–8) detector distances (*p* < 0.001). Models for the short detector distances (1 through 5) achieved a higher *R*^2^ than the averaged *R*^2^, indicating a higher predictability than those for the longer detector distance (6 through 8).

Given the importance of evaluating the red coloration of the whole flesh, we calculated the average anthocyanin content of the whole flesh based on the data of individual flesh depths and created the PLS models for the whole flesh. Figure 7 shows the average (± SE) *R*^2^ for the PLS models developed using interactances collected at different SDS distances. Significant differences were observed between short (1 through 5) and longer (6–8) SDS distances (*p* < 0.001). Models for the short SDS distances (1 through 5) achieved a higher *R*^2^ than the averaged *R*^2^, indicating a higher predictability than those for the longer SDS distance (6 through 8). Although there was no statistically significant difference between SDS distances 1 through 5, the models obtained at SDS distances 4 and 5 achieved the higher predictive accuracies than the shorter SDS distances 1–3. This suggests that a proper SDS distance could improve the predictivity of the models based on such an interactance spectroscopic approach.

#### One-way ANOVA of model accuracy for skin and individual flesh depths

**Figure 8:**
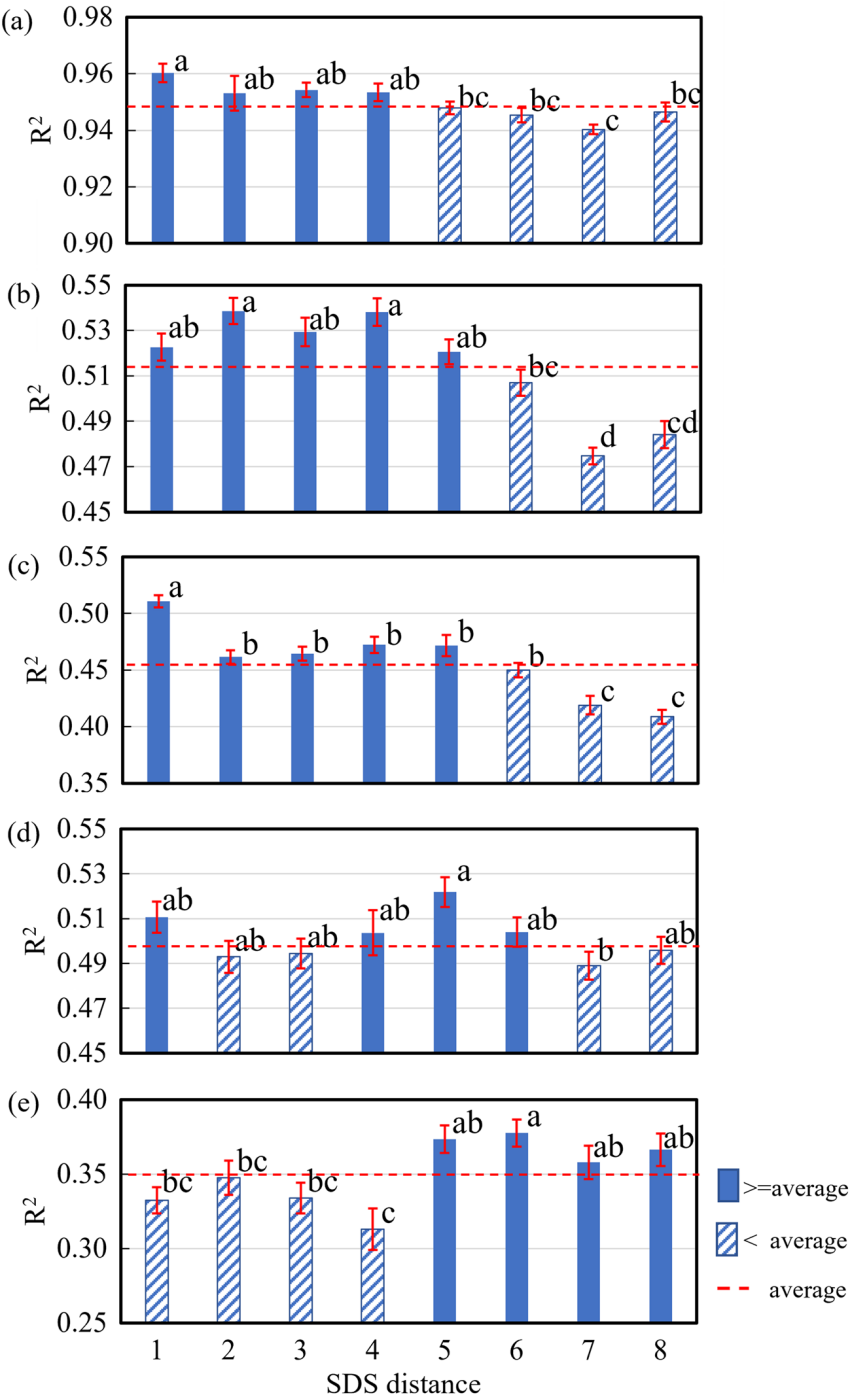
Average (± SE) *R*^2^ for PLS models for skin (**a**) and individual fleshes at different depths [Flesh 1 (**b**), Flesh 2 (**c**), Flesh 3 (**d**), and Flesh 4 (**e**)] based on interactances collected at different SDS distances. Significant differences were observed between certain SDS distances for both skin and fleshes at all depths, showing that the optimal SDS distance suited to estimate anthocyanin content in flesh at different depth may differ from one another.

Varied levels of significant differences were observed between certain SDS distances for both skin and fleshes at all depths (Fig. 8). Although there were no statistically significant differences between some of the detector distances, overall, models for short detector distances (1 through 4 for Skin, 1 through 5 for Flesh 1 to 2) achieved a higher *R*^2^ than the averaged *R*^2^ (Fig. 8a–c), indicating a higher predictability than those for the longer SDS distance. Conversely, models for longer SDS distances (4 through 6 for Flesh 3, 5 through 8 for Flesh 4) achieved a higher *R*^2^ than the averaged *R*^2^ (Fig. 8d,e), suggesting that the interactances collected at longer SDS distances might contain more information about anthocyanin content in the fleshes at deeper depths.

#### Optimal models for skin and whole flesh

**Figure 9:**
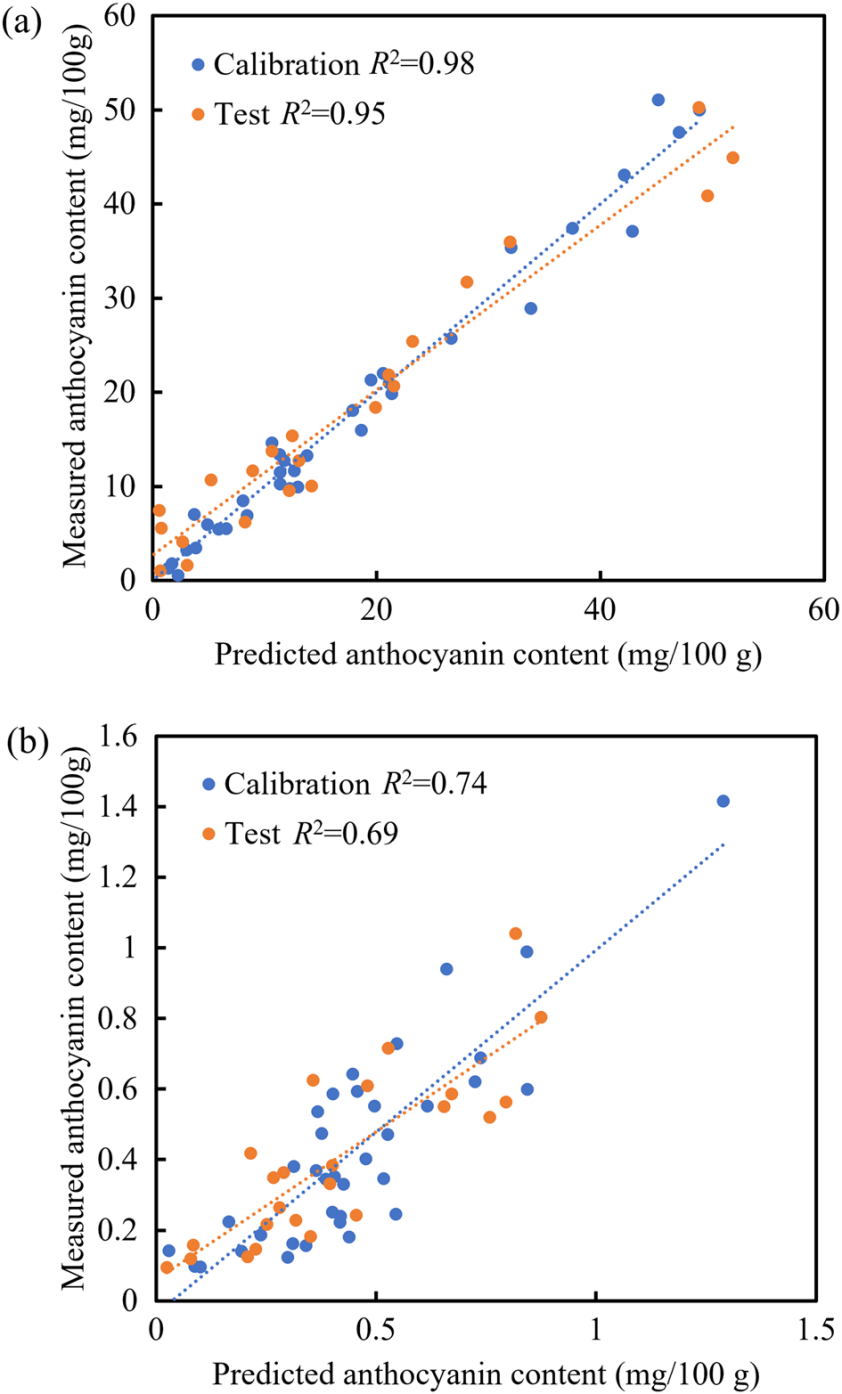
Predictive performance of the PLS models based on interactances obtained at the optimal SDS distances. The optimal PLS model for skin (**a**) was obtained at SDS distance number 1. The optimal PLS model for whole flesh (**b**) was obtained at SDS distance number 4.

The PLS models for skin and whole flesh based on the averaged interactances for the optimal SDS distances (detector’s specific positions number 1 for skin and number 4 for whole flesh) identified in the previous analysis were developed. Scatter plots of the estimated against the measured anthocyanin contents and the coefficients of determination (*R*^2^) for the predictions are illustrated in Fig. 9. The model for skin achieved satisfactory predictions for both the calibration and the test datasets (both *R*^2^ > 0.95) (Fig. 9a). The model for whole flesh also obtained a good predictive performance in the calibration (*R*^2^ = 0.74), and a reasonable predictive accuracy (*R*^2^ = 0.69) in the model test (Fig. 9b). These results indicated that it is easier to predict the anthocyanin content in the skin than the flesh of apple. This study also demonstrated the possibility of estimating the anthocyanin content in apple flesh from the interactance data obtained with our novel spatially resolved interactance spectroscopy system.

## Discussion

This study revealed the difficulty to discriminate the flesh color simply by visual inspection of the skin appearance of the ‘Kurenainoyume’ fruit. Statistical analyses further confirmed the poor correlations between anthocyanin content in skin and flesh. Empirically, the red coloration in skin depends on sunlight exposure and therefore the degree of pigment in skin can be controlled by physical measures such as bagging or rotating on-tree fruits to regulate the duration and amount of sunlight exposure in practices; nevertheless, the red coloration in flesh depends less on sunlight and its color formation during fruit development can continue until late autumn and cold season^42^. Genetical analyses revealed that ‘Kurenainoyume’ was a new type 2 red-fleshed apple cultivar^1,43^. In this type of apples, the coloration of skin and fruit flesh was controlled by different anthocyanin-activating genes. In ‘Kurenainoyume’ cultivar, *MdMYB10* (*MdMYBA*) and *MdMYB110a* were found to be the anthocyanin-activating genes responsible for the red coloration in skin and fruit flesh, respectively^44^. Due to this fact, the flesh color has proved difficult to predict from the skin appearance because their color formation during fruit development is controlled by two essentially different mechanisms. In addition, our results demonstrated significant differences in the red coloration between flesh layers at different depths in the same fruit. These results suggest the difficulty to get a full understanding of the red coloration in the whole flesh and therefore the necessity to develop a new technique to detect and discriminate the flesh color of red-fleshed apples.

Herewith, we developed a spatially resolved interactance spectroscopy system to estimate the degree of red coloration in the flesh of ‘Kurenainoyume’ apples. ANOVA analysis of the predictive accuracy (*R*^2^) of the PLS models for all individual flesh depths as well as the whole flesh showed that the models based on interactance spectra obtained at different SDS distances achieve different predictive accuracy for anthocyanin estimation and increasing the SDS distance to an appropriate extent tends to improve the predictive accuracy. In particular, the models for whole flesh obtained at the SDS distance 4, rather than the shorter SDS distances 1 through 3, achieved the highest predictive accuracy (Fig. 7). This might be attributed to the fact that more interactions occur between scattered light and fruit tissues when incident light passes through a longer distance within the flesh of a fruit, and the interactance spectra collected after these interactions provide more useful information about the internal properties of the fruit. However, a further increase in the SDS distance to the SDS distances 6–8 leads to reduced predictive accuracy. This is largely because the interactance becomes much weaker and less informative as most of the light is absorbed by the flesh and thus cannot reach the detector to be captured.

Moreover, in addition to the accurate model for the skin, the model for the whole flesh based on the interactance at the optimal SDS distance also achieved reasonable prediction results, which are much better than our previous work (both *R*^2^ for calibration and test were between 0.5 and 0.6)^21^. These results confirmed our hypotheses on the pathways of light transmission and interaction in fruit flesh. This fundamental principle underlying the light behaviors in fruit flesh provides justifications for further investigation and development of instruments for detecting the fruit flesh coloration in the future.

The findings of this study also suggest that the spatially resolved interactance spectroscopy system might be potentially applied to other similar fruits, because it provides the possibility of identifying an optimal SDS to obtain the interactances that are associated with the target ingredients in the fruits. However, the models presented in this study might not be simply applicable to them, because different fruits, even different varieties of the same fruit have different fruit structures, and the skin and flesh textures also differ among different fruits (varieties). It is necessary to test the transmissibility of the light emitted from light source within a particular fruit type. In addition, the use of an enhanced light source with a few specific wavelengths rather than a wide range of wavelengths would be another option when designing the instrument system. This necessitates the identification of specific wavelengths that are related to the target ingredients and contributable to the estimation of these ingredients. On the other hand, the pigments responsible for flesh coloration may also be different among different fruits (varieties). Therefore, the models for anthocyanin estimation in the “Kurenainoyume” apple variety based on a certain SDS distance may not simply apply to other fruits (varieties). There is a need to identify an optimal SDS distance and develop a calibration model for a specific fruit (variety). The above-mentioned issues will be further studied in our future work.

This study demonstrated the possibility of estimating the anthocyanin content in apple flesh from the interactance data obtained with our novel spatially resolved interactance spectroscopy system. By identifying an optimal SDS distance for the measurement system it is possible to assess the internal quality of red-fleshed apples with reasonable estimation accuracy. This new system may be potentially applied to grading and sorting systems for red-fleshed apples in fruit industry.

## Methods

### Fruit material and sample preparation

‘Kurenainoyume’, a red-fleshed apple cultivar, was used in this study. The fruit is sweet, mildly tart, rich in anthocyanin, and is delicious either fresh or cooked. The natural red color of flesh remains distinct even after cooking or processing, making it particularly useful for the creation of richly colored apple products^45^. Therefore, it is welcomed by both consumers and manufacturers of apple fruit products.

Trees of this precious cultivar were cultivated in Fujisaki Farm of Hirosaki University. The fruits were harvested in a timely manner and carefully handled during harvest and transport. After screening them to ensure a high degree of fruit size uniformity, a total of 30 fruit samples were selected for the experiment. As the skin color on different sides of a fruit largely depends on whether the side is exposed or unexposed to direct sunlight, the skin on different sides is not always uniform in color. Therefore, for each fruit sample, two spots with maximum color difference (the darkest and lightest areas of skin color) on the fruit surface, which are usually located on opposite sides of a fruit (exposed and unexposed to direct sunlight), were selected for subsequent interactance measurement and anthocyanin quantification. As a result, a total of 60 data samples for each measurement or quantification in the following experiments were obtained for further analysis. All plant studies (fruits of ‘Kurenainoyume’) were carried out in accordance with relevant institutional, national or internal guidelines and regulation.

### Spatially resolved interactance measurement system

**Figure 10:**
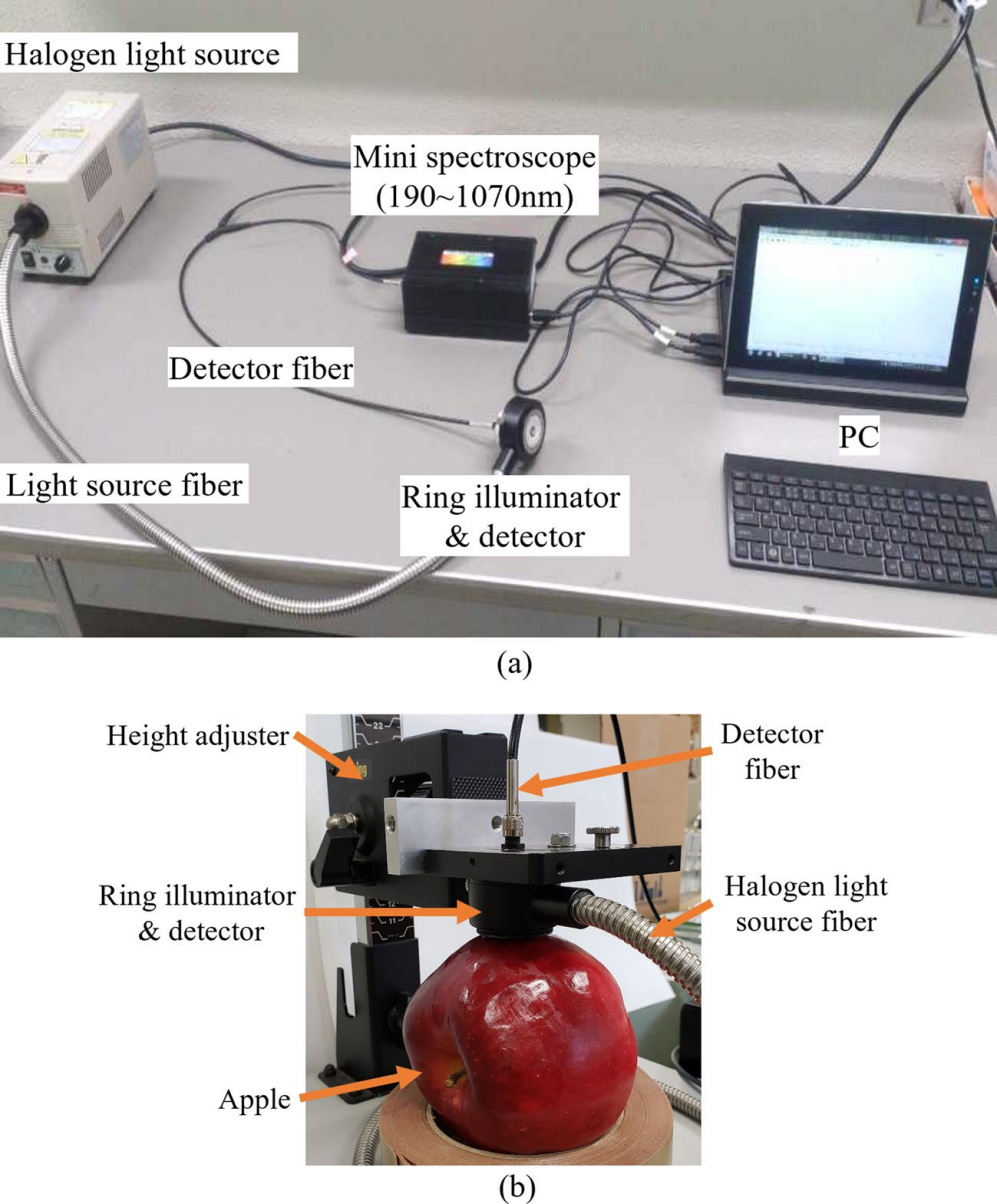
Instruments and experimental setup of the spatially resolved interactance spectroscopy system. Instrument components (**a**) and interactance measurement of a sample (**b**) with the system.

A spatially resolved interactance measurement system was developed for collecting interactance spectra for apple fruits. The system is composed of a halogen light source generator LA-150ue-A (Hayashi Co., Japan), a ring illuminator (Hayashi Co., Japan) integrated with a self-made movable detector fiber, and a mini-spectroscope BLACK-Comet-SR100 (StellarNet Inc., USA) (Fig. 10a). During measurement, the ring illuminator was tightly placed on the fruit surface (Fig. 10b), allowing the halogen light to pass through the fruit skin and enter the flesh, and the amount of light that returned to the detector after scattering in the flesh was measured.

The mini spectroscope covers ultraviolet, visible and near infrared spectroscopic wavelengths from 190 to 1070 nm. The mini spectroscope is connected to a computer, and the interactance measurements for the sample are recorded when the system is operated by the software SpectraWiz (StellarNet Inc., USA) installed on a PC. Once the data is recorded, the interactance spectra for each measurement can be exported with the software for further analysis.

**Figure 11:**
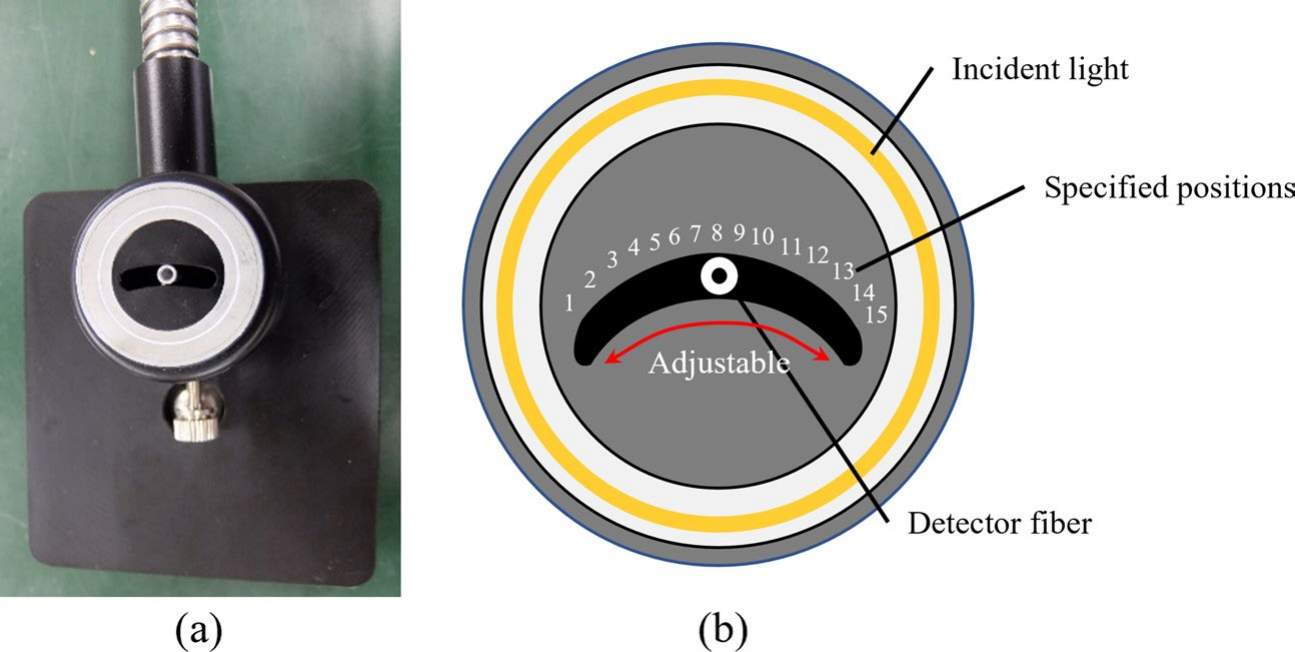
Ring illuminator and detector (**a**) and its schematic diagram (**b**) used to acquire interactance measurements. The ring-type design of the illuminator enhances the intensities of incident light and interactance collected by the detector. The detector is movable between preset positions from number 1 to 15, and the distance between any two adjacent positions is constant (2 mm). The illustration in the schematic diagram (**b**) was made using Microsoft PowerPoint for Microsoft 365 (https://www.microsoft.com/ja-jp/microsoft-365/powerpoint).

The working principle of the above system is described in more detail below. Figure 11a shows the structure of the ring illuminator integrated with the detector. The light from the halogen light source generator enters the ring illuminator through connecting fibers and forms a ring-type light beam (yellow ring) (Fig. 11b). The outer black cover and the inner light shield (black area) block the light that is directly reflected from fruit surface (Fig. 11b). The hole in the center serves as an entrance slit for the light that comes back to the detector after scattering in the flesh and passing back through the fruit skin (Fig. 11ab). In this structure, the light source (ring-type beam) and detector (entrance slit) are positioned parallel to each other, thus light due to specular reflection cannot directly enter the detector. The fibers leading to the source and detector are parallel to each other and in contact with the product (Fig. 10a).

Furthermore, in this structure, the detector is designed to be movable rather than being fixed in the center point of the device. This enables the device to obtain spatially resolved interactance spectra from apple fruits. The new device could obtain interactance spectra with the detector, whose distance from light source could be adjusted to eight specified SDS distances. In Fig. 11b, the detector is placed in the center (number 8), which has the farthest SDS distance, and the numbers 1 to 15 represent different positions of the detector at eight different SDS distances (Except for the number 8 of the specific positions (the center of the device), every two of other specific positions, such as numbers 1 and 15, numbers 2 and 14, etc., have the same distance from the light source, therefore the device has a total of 8 different SDS distances). Such a movable detector instead of multiple detectors placed at different positions on samples (Fig. 1) might also acquire spatially resolved interactance needed for analysis of internal properties of fruit flesh at different depths.

### Quantification of anthocyanin contents in apple flesh

In addition to the skin, anthocyanins are present in large amounts in the flesh of red-fleshed apples. They can be increased up to several 100-fold in red flesh compared with white flesh^46^. Therefore, the anthocyanin content was used as an indicator for the degree of red coloration in the flesh.

**Figure 12:**
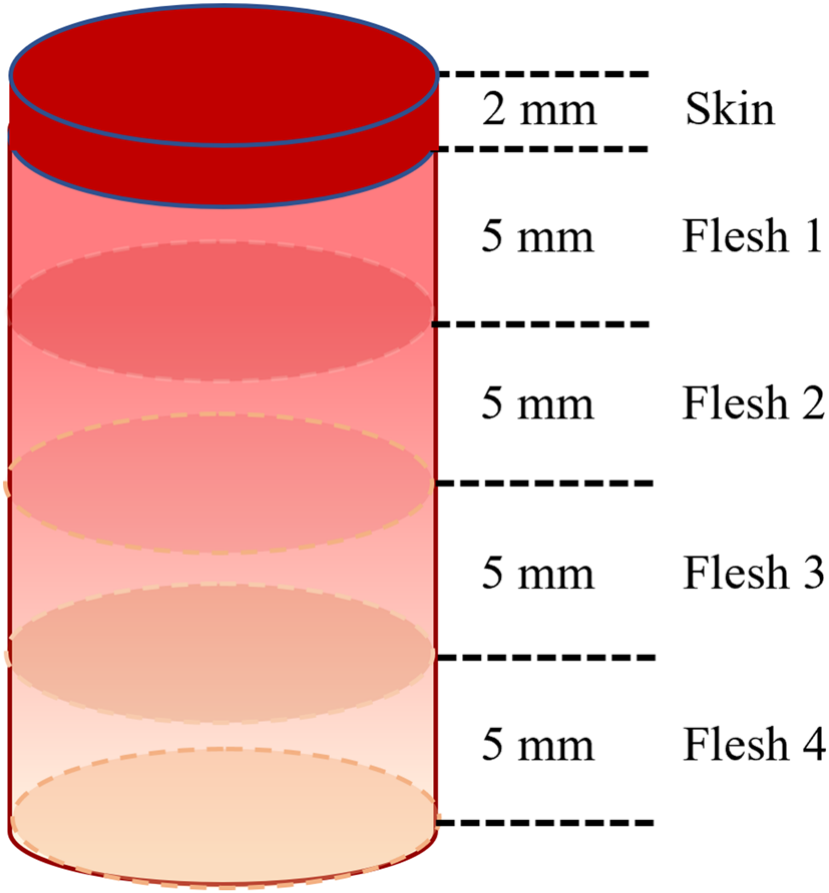
Graphical illustration of skin and fleshes at different depths used for anthocyanin quantification.

After the preceding interactance measurements, part of the fruit including the skin and the flesh under the measured position was prepared for quantifying anthocyanin content. A cork borer (diameter: 20.5 mm) was first used to obtain cylinders of tissue from the measured position in the fruit. Fruit skin with a thickness of 2 mm, and 4 pieces of the flesh under the skin, each with a thickness of 5 mm, were cut separately along the flesh depth, as illustrated in Fig. 12. Anthocyanins were extracted using the conventional method with polar solvents^47^. Each of the cut skin and flesh pieces was weighed and then cut into two halves. The prepared samples were put into tubes and 10 ml of 0.1% hydrochloric acid in methanol was subsequently added into the tubes and kept in a refrigerator (4–8 °C) up to 24 h for pigment extraction. After the extraction, the absorbance of the resulting solution containing anthocyanins were measured at 530 nm with a SHIMADZU UV-1800 spectrophotometer (Shimadzu Inc., Kyoto, Japan), based on which the anthocyanin contents in the samples were calculated by the equation in the literature^48^.

### Data analysis and model development

The spatially resolved interactance spectra were used to relate to the anthocyanin content in skin and flesh at each depth. The partial least squares (PLS) regression, a standard calibration method for analyzing spectral data^49^, was used to develop the prediction models. We used the R package ‘pls’ for modeling analysis^50^. In the modelling, we employed the leave-one-out method for cross validation, in which potential models are calculated by excluding only one observation at a time^51^.

In the measurement of spatially resolved interactance, except for number 8 of the detector’s specific positions (the center of the device), every two of other specific positions, such as numbers 1 and 15, numbers 2 and 14, etc. (Fig. 11b), have the same separation distance from the light source, therefore the averaged interactances of these paired positions were calculated. As a result, the averaged interactances of eight different SDS distances were used to develop the PLS models. The entire data set was randomly divided into two subsets, one used for model calibration (60% of data) and the other for model test (40% of data). As the random selection of data results in different data subsets for model calibration and test each time, the data subsets obtained by a single selection could not accurately reflect the internal relationship between the data variables. Therefore, we repeated the process of random data selection and subsequent model calibration 50 times and created 50 PLS models for each data set. The coefficient of determination (*R*^2^), a standard metric to evaluate regression analyses^52^, was calculated for the model test dataset and was used as an indicator for each model’s predictive accuracy.

To compare the predictive accuracy of the models developed, three types of analysis of variance (ANOVA) were conducted as follows: firstly, a two-way ANOVA was conducted to examine the main and interactive effect of SDS distance and flesh depth on model predictive accuracy (*R*^2^); Secondly, a one-way ANOVA was conducted to identify the optimal SDS distance for estimating the averaged anthocyanin content in the whole flesh; Thirdly, one-way ANOVAs were conducted for skin and each flesh depth individually to examine the effect of SDS distance on model’s accuracy for skin and individual flesh depths. Finally, the optimal PLS models for estimating anthocyanin content in the skin and the whole flesh were developed using the optimal SDS distances identified in the ANOVAs.
